# Hybrid Capture-based Genomic Profiling of Circulating Tumor DNA From Patients With Advanced Ovarian *Cancer*

**DOI:** 10.3389/pore.2021.581534

**Published:** 2021-04-09

**Authors:** Wenbin Shen, Boer Shan, Shanhui Liang, Junling Zhang, Yangyang Yu, Yuzi Zhang, Guoqiang Wang, Yuezong Bai, Bing Qian, Jin Lu, Zhi Jiang

**Affiliations:** ^1^ Department of Oncology, Fudan University Shanghai Cancer Center, Shanghai, China; ^2^ The Medical Department, 3D Medicines Inc., Shanghai, China; ^3^ Departments of Gynecomatous Surgery, Jiangsu Cancer Hospital, the Affiliated Hospital of Nanjing Medical University, Nanjing, China

**Keywords:** circulating tumor DNA, liquid biopsy, ovarian cancer, genomic profiling, genomic alterations

## Abstract

**Objective:** We conducted this study to characterize somatic genomic alterations in circulating tumor DNA (ctDNA) from patients with ovarian cancer and compare GAs detected in ctDNA with tissue databases.

**Methods:** Hybrid capture-next generation sequencing genomic profiling of 150 genes was performed on ctDNA from 138 patients with ovarian cancer with 1,500× sequencing depth. The GAs detected in ctDNA were compared with those in our ovarian cancer tissue database (N = 488) and the *Cancer* Genome Atlas (TCGA) database (N = 489).

**Results:** 115 patients (83%) had at least 1 GA detected in ctDNA. The most frequently altered genes detected in ctDNA were *TP53* (72%), *KRAS* (11%), *LRP1B* (10%), *ZNF703* (9%) and *NF1* (8%). Comparative analysis with our tissue database showed similar frequencies of GAs per gene, although *PIK3CA* and *KRAS* mutations were more frequent in tissue and ctDNA, respectively (*p* < 0.05). Gene amplification and rearrangement were more frequent in ctDNA samples. The mutation frequency of homologous recombination repair associated-genes, VEGF signal/angiogenesis pathways, RAS pathways, NOTCH pathways and MSI-H ratio was not statistically different either in ctDNA or in tissue database. However, the mutation frequency of *AKT*, *PIK3CA*, *PTEN* and *STK11* in PI3K/AKT/mTOR pathway was significantly lower than that in tissue samples (*p* < 0.05).

**Conclusions:** Our results suggest that genomic profiling of ctDNA could detect somatic GAs in a significant subset of patients with ovarian cancer. Hybrid capture-NGS based on liquid biopsy has the potential capability to serve as a substitute to tissue biopsy and further studies are warranted.

## Introduction

Ovarian cancer is the leading cause of cancer-related death among gynecological cancers and the standard treatment for primary disease is cytoreductive surgery of the tumor followed by adjuvant chemotherapy, platinum and taxane-combined treatment. However, clinical data suggest that more than half of ovarian cancer patients develop chemotherapy resistance and recurrent disease [1]. Therefore new therapeutic regimens including targeted therapy with bevacizumab or the poly ADP-ribose polymerase (PARP) inhibitor olaparib have presented promising clinical activity. Patients with *BRCA1/2* mutations are the primary beneficiaries of PARP inhibitors, followed by homologous recombination repair (HRR) [2]. For persistent/recurrent patients, it is recommended that tumor molecular detection of *BRCA1/2*, dMMR/MSI-H and *NTRK1/2/3*, be performed prior to initiation of therapy [2]. Therefore, an effective prediction of sensitivity and resistance to targeted therapies for ovarian cancer is key to precision medicine, in which genomic alterations (GAs) are crucial for targeted therapies for ovarian cancer [3].

Currently, genomic detection based on tissue tumor specimens remains the standard for genetic testing. However due to some clinical risks and surgical complications, as well as the specificity of the mechanism of ovarian cancer metastasis, a considerable number of patients do not receive tissue biopsy. The metastasis of ovarian cancer is known to develop in two completely different pathways. Although the primary mechanism for metastasis of ovarian cancer has long been the passive dissemination of the tumor globule through the peritoneal fluid and ascites, hematogenous metastasis of circulating cancer cells has subsequently been found to be the preferred route to the omentum [4]. Blood-based genomic analysis provides a non-invasive alternative to traditional biopsy, has the advantage of detecting heterogeneous changes in metastatic lesions, and may provide complementary genomic information for tissue-based detection. The consistency of tumor and ctDNA in different GAs has not been reported in large populations of ovarian cancer. Furthermore, it remains unclear whether the GAs frequencies found in ctDNA are similar to those reported in the large tissues based on the next generation sequencing (NGS) studies.

We conducted a retrospective study of hybrid capture-based NGS genomic profiling of 150 genes panel on ctDNA from patients with ovarian cancer. We compared somatic alterations detected in ctDNA with our tissue database and the *Cancer* Genome Atlas (TCGA) database.

## Materials and Methods

### Samples

Blood samples were obtained from 138 patients with ovarian cancer between January 2017 and January 2020, and ctDNA sequencing was performed on Illumina HiSeq sequencer (Illumina, San Diego, CA) with a median unique exon coverage depth of 1,500× in a College of American Pathologists (CAP) and Clinical Laboratory Improvement Amendments (CLIA) certified laboratory (3D Medicine Inc: Shanghai, China) [5]. Hybrid capture-based NGS genomic profiling of a well-designed 150 cancer gene panel was performed on ctDNA. Somatic alterations were identified and clinical information including age, gender, and tumor histology was collected. A waiver of informed consent form was signed by each patient, and the study was approved by the ethics committee of the hospital.

### DNA Extraction

Briefly, 20 ml of peripheral whole blood was collected for genomic profiling of ctDNA. The blood was centrifuged in Streck tubes at 1,600 g for 20 min at room temperature to separate the plasma. Then, the plasma layer was carefully transferred to a new 1.5 ml Eppendorf tube, followed by room-temperature centrifugation at 16,000 *g* for 10 min to remove residual cells and debris. The buffy coat was then transferred to a new tube for genomic DNA (gDNA) extraction. The QIAamp Circulating Nucleic Acid Kit (Qiagen) was used to extract ctDNA from the plasma, respectively, following the standard protocols, and then fragmented to a size ranging from 200 bp to 400 bp using Covaris S2 SonoLAB (Covaris). DNA concentrations were determined by the Qubit dsDNA HS Assay Kit (LifeTechnologies).

### Library Preparation and ctDNA Sequencing

The assay methodology and procedure were as previously described [5]. Briefly, 20–100 ng of cfDNA was extracted to create adapted sequencing libraries. The ctDNA libraries were prepared by the Accel-NGS 2 S Plus DNA Library Kit (SWIFT) with unique identifiers (UIDs, also called barcodes) to tag individual DNA molecules. The captured DNAs were then amplified by PCR, and the final DNA concentrations and sizes were respectively measured by Qubit and Caliper. The captured libraries for FFPE gDNA were loaded into the Illumina HiSeq sequencer. The fraction of ctDNA was estimated using the maximum somatic allele frequency (MSAF). GAs including single nucleotide variation (SNV), insertions/deletions, copy number variations (CNV) and gene fusions were assessed and the corresponding criteria are the same as our previous study [6]. Germline alterations were excluded. We included in this study only samples with a tumor cell percentage >20.

### Statistical Analysis

The paired-end reads were mapped by BWA [7] MEM algorithm. SNVs were called by MuTect [8] with default parameters. Small insertions and deletions were called from the union of Varscan 2 [9] and Pindel [10] with default parameters. Fusions were called by selfdeveloped scripts with at least 5 pairs of reads spanned over the breakpoints between two partner genes. The CNVs of tumor tissues were calculated by BIC-seq2 [11] with default parameters, and the CNVs of ctDNA samples were called by a method reported by Jacob J. Chabon et al. [12]. All mutations were manually reviewed using integrative genomics viewer (IGV) [13] to further eliminate falsepositive results. The probability density distributions of mutant and wild-type fragments were calculated by Gaussian kernel smoothing using StatsModels 0.8.0.

Categorical variables were described as number and proportions. Categorical relationships were examined by using Pearson’s chi-square test with the Yates continuity correction when applicable and *p* value <0.05 was considered statistically significant. The SPSS22.0 software (SPSS, Inc., Chicago, IL, USA) was carried out for statistical analysis.

## Results

### Patient Characteristics

Hybrid capture-based genomic profiling was performed on plasma samples collected from 138 patients with ovarian cancer. The baseline characteristics for the patients were described in Table 1. In brief, the disease histology of ovarian cancer was ovarian serous cystadenocarcinoma (100%). The median age was 57 years.

**TABLE 1:** Characteristics of ctDNA samples.

Characteristic	All cases
Cases, n	138
Median age, y (range)	57 (31–81)
Stage, n (%)	
Ⅰ	16 (11.6%)
Ⅲ	52 (37.7%)
Ⅳ	70 (50.7%)
Histology, n (%)	
Ovarian serous cystadenocarcinoma	138 (100%)
Grade, n (%)	
1	2 (1.4%)
2	4 (2.9%)
3	101 (73.2%)
NA	31 (22.5%)
MSAF >0 (%)	115 (83%)
Median MSAF	0.026 (0.0003–0.2822)
Avg. GA/case^a^	3.83

^a^ Includes only cases with MSAF >0.*G1, well-differentiated; G2, moderately differentiated; G3, poorly differentiated; NA, Not Available.

### Distribution of GAs Identified in ctDNA

The MSAF was determined as the maximum allele frequency for all the mutations detected per sample in blood. The MSAF greater than zero was used as the evidence of ctDNA in the blood and ctDNA was detected in 115 (83%) samples. The median MSAF across all cases was 0.026 (range, 0.0003–0.2822), and among cases with evidence of ctDNA present, the average of reportable GA was 3.83 GA/case (Table 1).

**FIGURE 1:**
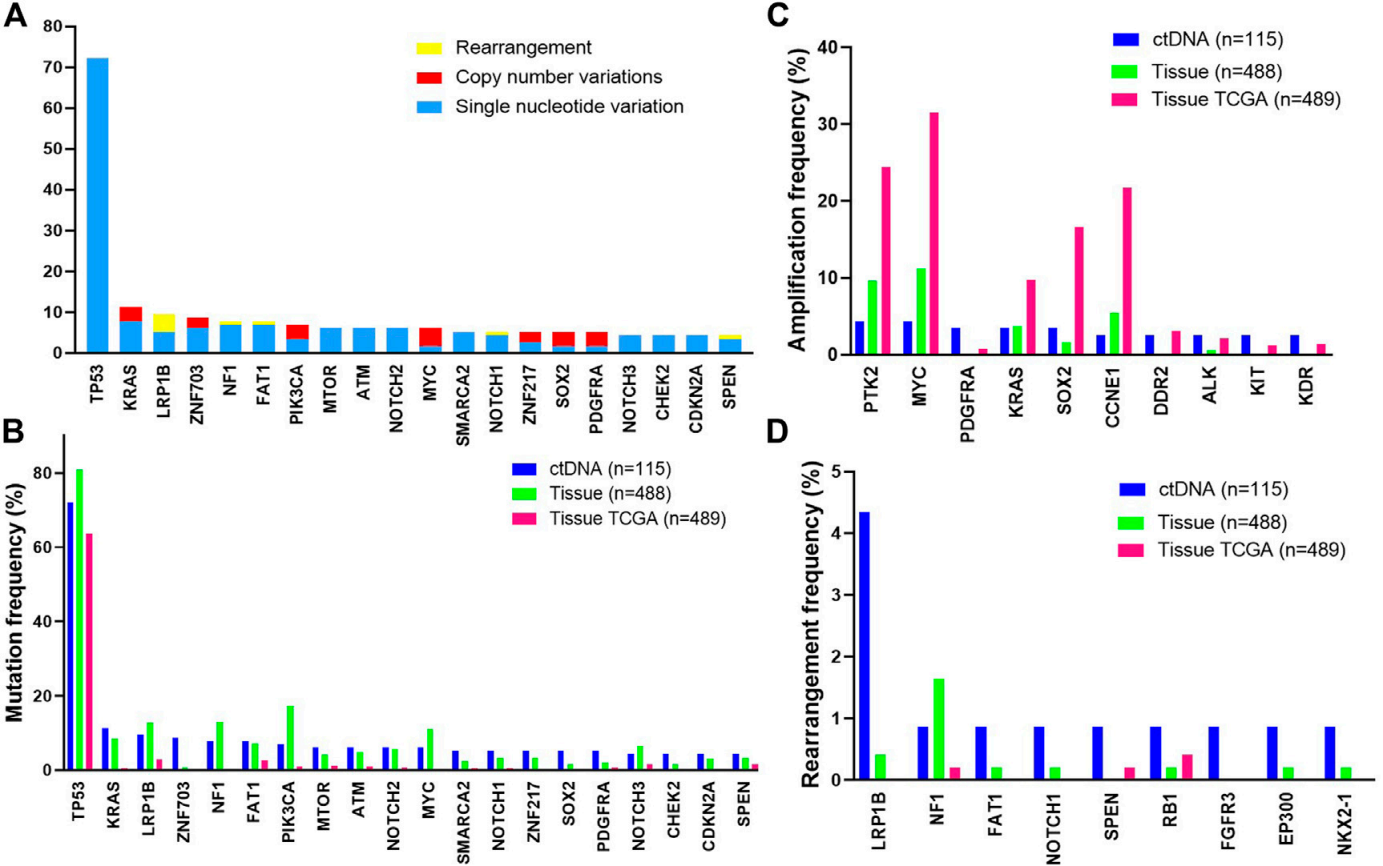
The most frequent genomic alterations identified in circulating tumor DNA (ctDNA) from patients with ovarian cancer vs. in tissue. Samples with evidence of ctDNA in the blood (maximum somatic allele frequency >0) are included **(A)** Longtail of frequently altered genes in ovarian cancer. B-D. Comparison of the most frequently mutated **(B)**, amplified **(C)**, or rearranged **(D)** genes observed in ctDNA in this study with tissue-based genomic profiling of ovarian cancer cases or with a published tissue-based genomic profiling study of ovarian cancer (The *Cancer* Genome Atlas [TCGA], 2011). Data from the TCGA study were extracted from the cBioPortal.

Among 115 cases with evidence of ctDNA, the most frequently altered genes were *TP53* (72%), *KRAS* (11%), *LRP1B* (10%), *ZNF703* (9%) and *NF1* (8%) (Figure 1A). We further compared common GAs detected in ctDNA with those detected in tissue samples from patients with ovarian cancer and with those from TCGA [14]. The frequencies of common GAs were largely similar between ctDNA and tissue samples in our tissue database for patients with ovarian cancer, while different from TCGA (Figure 1B). These included *TP53* (72% vs. 81% vs. 63.8%), *KRAS* (11% vs. 9% vs. 0.41%), *LRP1B* (10% vs. 13% vs. 2.9%), *ZNF703* (9% vs. 1% vs. 0%), *NF1* (8% vs. 13% vs. 4%) and so on. The gene amplification detected in ctDNA samples was inconsistent with our tissue samples. The most commonly detected gene amplification in of blood was *PTK2*. The detected amplification rates of *PDGFRA* (3.5% vs. 0%), *DDR2* (2.6% vs. 0.2%) and *KIT* (2.6% vs. 0.2%) were higher in blood than tissue (*p* < 0.05), while the amplification rate of *MYC* was lower in blood than tissue (4.3% vs. 11.3%, *p* = 0.02, Figure 1C). Gene rearrangement was more frequently detected in ctDNA samples from this study than in tissue samples (Figure 1D).

**FIGURE 2:**
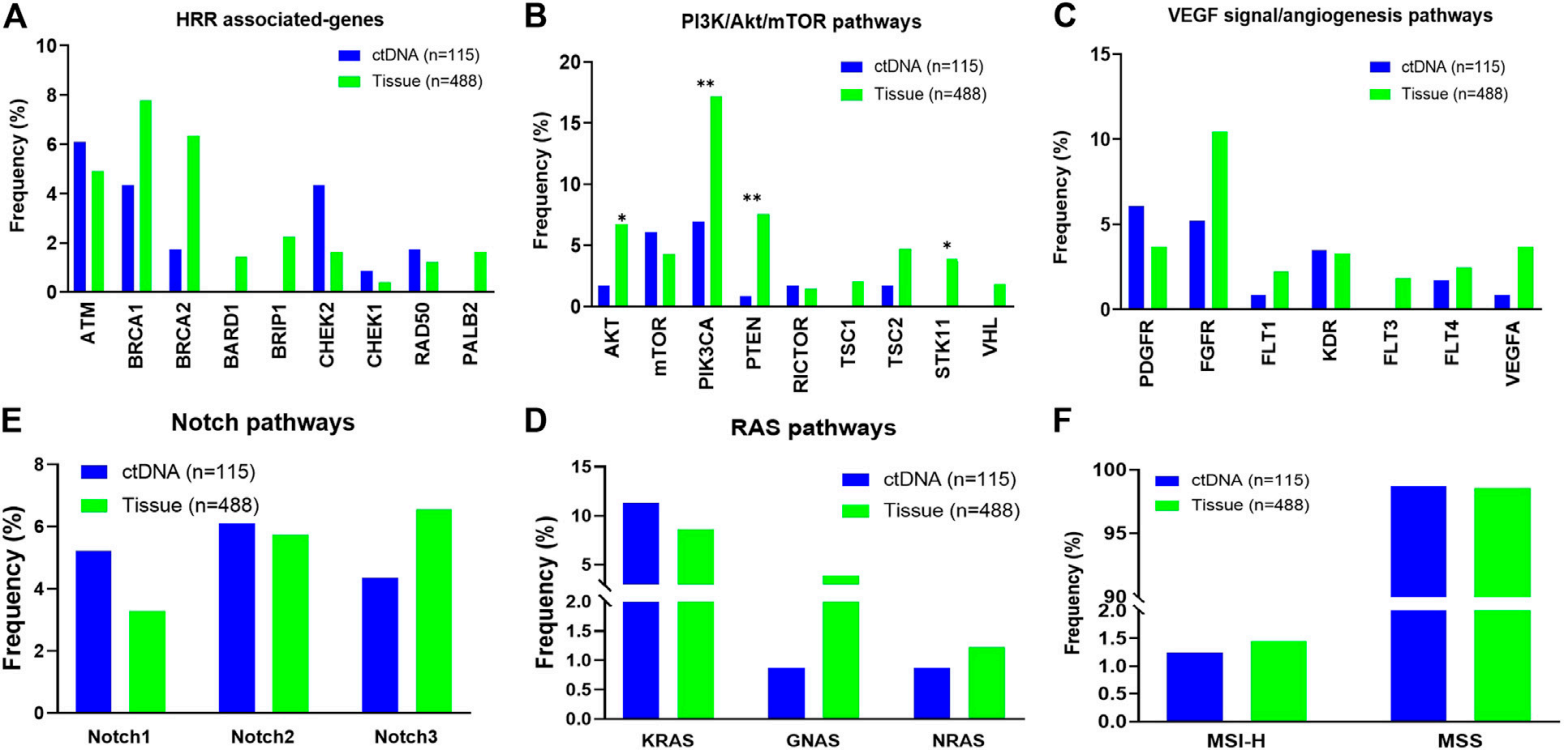
Distribution of individual gene alterations within pathways identified in circulating tumor DNA (ctDNA) from patients with ovarian cancer vs. in tissue. **(A)** HRR associated-pathways. **(B)** PI3K/AKT/mTOR pathways. **(C)** VEGF signal/angiogenesis pathways. **(D)** RAS pathways. **(E)** NOTCH pathways. **(F)** The ratio of dMMR/MSI-H.

Potentially actionable GAs informing selection of matched targeted therapies and clinical trials or predicting lack of response to antibody therapies were identified. GAs of *BRCA1/2* (HRR associated-genes) were observed in 6.1% of ctDNA cases and 14.1% of tissue cases. Somatic SNV alterations in other HRR associated-genes including *ATM*, *CHEK1/2* and *RAD50* were detected in 6.1%, 5.2% and 1.7% of patients in ctDNA compared to 4.9%, 2.0% and 1.2% of patients in the tissue database (Figure 2A). In addition, a total of 26.1% of cases harboring at least one alteration in PI3K/AKT/mTOR pathways in ctDNA sample (vs. 34.5% in tissue sample, Figure 2B). And 13.0% of ctDNA samples showed at least one VEGF signal/angiogenesis pathways associated-genes mutations, compared with 20.0% in the tissue samples. However, the mutation frequency of *AKT*, *PIK3CA*, *PTEN* and *STK11* in PI3K/AKT/mTOR pathway was significantly lower than that in tissue samples (*p* < 0.05, Figure 2C). The mutation frequencies of RAS pathways (12.2% vs. 12.7%) and NOTCH pathways (13.9% vs. 14.1%) were similar between ctDNA and tissue. (Figures 2D,E). In addition, we found the ratio of MSI-H was not different in ctDNA and tissue samples (1.3% vs. 1.4%, Figure 2F).

## Discussion

In this study, we have reported results of hybrid capture-based NGS of 138 ovarian cancer ctDNA samples. ctDNA was detected in 83% of samples, which is similar to the rate in ovarian cancer in other recent reports [15]. Among cases with evidence of ctDNA, an average of 3.83 reportable alterations per case was detected. A total of 26.1% of the cases showed at least one change in the PI3K/AKT/mTOR pathways. A total of 17.4% of cases harbored at least one alteration in HRR associated-genes. Deleterious mutations in *BRCA1/2* were detected in 6.1% of cases.

Furthermore, we compared common GAs detected in ctDNA with those detected in tissue samples from patients with ovarian cancer and with those from TCGA. Almost every gene mutation tested in our tissue database could be found in ctDNA data. And among the detected GAs, most genes had similar frequencies in both the ctDNA samples and the tissue samples, while different from TCGA. Tumor stage and sequencing depth are probably reasons for this difference. Overall, our findings had indicated that there was a certain concordance rate of genomic alterations in both tissue and ctDNA samples of ovarian cancer.

Several studies had found that gene amplification could predict overall survival in ovarian cancer [16]. The frequency of gene amplification detection in ctDNA samples was significantly lower than that detected in 3D tissue sample and TCGA database, similarly with published researches in colorectal carcinoma [17]. In contrast, the profile and incidence of the short variant alterations was similar to that observed in tissue samples from ovarian cancer patients, supporting the ability of ctDNA analysis to reflect tissue-based characteristics. Somatic genomic rearrangements are widespread in cancer genomes and might result in the structural variants. In our results, gene rearrangement was found in 4.3% of ctDNA samples. The frequency of ctDNA rearrangement is not particularly similar to that of tissue samples, as ctDNA fragments in the blood are likely to be shorter and tumor levels much lower than in tissue samples. Together, our data indicated that gene amplification and rearrangement could be effectively detected by ctDNA, which might provide a reference for targeted therapy.

PARP inhibitors have recently been approved to treat the advanced ovarian cancer patients with *BRCA1/2* mutations [2]. In our results, the incidence of *BRCA1/2* in ctDNA samples was 6.1%, lower than in tissue samples. However, studies have found that fluid biopsy is almost 100% sensitive to stage IV ovarian tumors [18]. Combining with our results, it indicated that ctDNA sequencing may aid the selection of ovarian cancer patients for PARP inhibition targeted therapy. Besides *BRCA1/2*, other HR deficiency genes such as *ATM, RAD50* and *CHEK1/2* were also analyzed in our study. Most HRR genes had a higher frequency of GAs in ctDNA than tissue. Alterations of HRR deficiency genes detected by ctDNA could be regarded as a complement to reflect HRR deficiency.

The limited availability of tumor tissue in advanced ovarian cancer presents a major clinical challenge. In cases where biopsy is prohibited, blood-derived ctDNA may provide an alternative method for genomic analysis, and ctDNA testing may have additional advantages in identifying heterogeneous alterations not present at a single tumor site. However, further research, particularly in patients with matched tissue and ctDNA samples, is needed to validate the clinical significance of ctDNA.

In conclusion, NGS-based ctDNA testing in ovarian cancer may provide a valuable alternative or complement to tissue testing, particularly in cases in which tissue biopsy is prohibitive or repeat genomic assessment in the setting of disease progression is indicated.

## Data Availability

The original contributions presented in the study are included in the article/Supplementary Material, further inquiries can be directed to the corresponding author.
